# Mass spectrometry based metabolomics for in vitro systems pharmacology: pitfalls, challenges, and computational solutions

**DOI:** 10.1007/s11306-017-1213-z

**Published:** 2017-05-19

**Authors:** Stephanie Herman, Payam Emami Khoonsari, Obaid Aftab, Shibu Krishnan, Emil Strömbom, Rolf Larsson, Ulf Hammerling, Ola Spjuth, Kim Kultima, Mats Gustafsson

**Affiliations:** 1 Department of Medical Sciences, Clinical Chemistry, Uppsala University, Uppsala, Sweden; 2 Department of Pharmaceutical Biosciences, Uppsala University, Uppsala, Sweden; 3 Department of Medical Sciences, Cancer Pharmacology and Computational Medicine, Uppsala University, Uppsala, Sweden; 4 Science for Life Laboratory, Uppsala University, Uppsala, Sweden

**Keywords:** Metabolomics, Mass spectrometry, Data handling, Batch effects, Drug metabolism

## Abstract

**Introduction:**

Mass spectrometry based metabolomics has become a promising complement and alternative to transcriptomics and proteomics in many fields including in vitro systems pharmacology. Despite several merits, metabolomics based on liquid chromatography mass spectrometry (LC–MS) is a developing area that is yet attached to several pitfalls and challenges. To reach a level of high reliability and robustness, these issues need to be tackled by implementation of refined experimental and computational protocols.

**Objectives:**

This study illustrates some key pitfalls in LC–MS based metabolomics and introduces an automated computational procedure to compensate for them.

**Method:**

Non-cancerous mammary gland derived cells were exposed to 27 chemicals from four pharmacological classes plus a set of six pesticides. Changes in the metabolome of cell lysates were assessed after 24 h using LC–MS. A data processing pipeline was established and evaluated to handle issues including contaminants, carry over effects, intensity decay and inherent methodology variability and biases. A key component in this pipeline is a latent variable method called OOS-DA (optimal orthonormal system for discriminant analysis), being theoretically more easily motivated than PLS-DA in this context, as it is rooted in pattern classification rather than regression modeling.

**Result:**

The pipeline is shown to reduce experimental variability/biases and is used to confirm that LC–MS spectra hold drug class specific information.

**Conclusion:**

LC–MS based metabolomics is a promising methodology, but comes with pitfalls and challenges. Key difficulties can be largely overcome by means of a computational procedure of the kind introduced and demonstrated here. The pipeline is freely available on www.github.com/stephanieherman/MS-data-processing.

**Electronic supplementary material:**

The online version of this article (doi:10.1007/s11306-017-1213-z) contains supplementary material, which is available to authorized users.

## Introduction

In vitro systems pharmacology involves studies on the impact of drugs or other chemicals, either alone or in combination, on the systemic profile of various in vitro models. This discipline is already well established at the level of transcriptomics, with the “Connectivity Map” (CMap) as an outstanding example (Lamb et al. 2006). Regarding the level of metabolomics, a few pilot studies using nuclear magnetic resonance spectroscopy (NMRS) have been reported (Aftab et al. 2014; Tiziani et al. 2011). The metabolome is particularly attractive in this context, as it can provide information at a molecular scale, which is more tightly connected with cellular function and responds faster to external changes than the transcriptome.

Recent significant technological advances in liquid chromatography (LC) and mass spectrometry (MS) have made current LC–MS systems able to quantify complex mixtures of low molecular weight agents (<1500 Da) across a wide concentration range, thereby enabling LC–MS based metabolomics. However, as the resolution of the sample content increase, so does the resolution of the contaminants present within the sample. This is why genuinely sensitive measurement technologies like LC–MS typically carry increased experimental related variability. Such variability may arise from contamination from solvents or tubes used in the laboratory setting as well as from the experimental setup. An in vitro LC–MS experiment is a time demanding procedure that often requires the samples to be assembled into batches (either sample preparation batches, as used here or analysis batches). Notably, handling and comparing cells at different phases may also increase variability. Samples could further have been collected at various time points, seasons of the year and could also have been stored and transported for different amounts of time (Kohler et al. 2016; Kirwan et al. 2012; Teahan et al. 2006). Any of these sources of diversity will give rise to unwanted variation and biases in the recorded data. Variability may also pertain to the LC–MS instrumentation itself, such as intensity/sensitivity decaying throughout the analysis, caused by aggregated components in the column, carry over effects in between samples, as well as saturation effects when the instrument is unable to concurrently process all molecules entering the MS instrument (Teahan et al. 2006; van der Kloet et al. 2009). Some of these issues can be reduced by routine maintenance of the LC–MS equipment. However, this will not necessarily return the instrument to its initial performance.

In order to make correct interpretations of MS spectra, the issues presented above have to be adequately managed. However, although these issues are well known within the LC–MS field, there is yet no reported commonly accepted best practice procedure available, which helps achieving the desired suppression of externally caused variability. Several published LC–MS based metabolomic studies, not necessarily involving chemically induced changes in vitro as studied here, have addressed these issues (Beckner Whitener et al. 2016; Ganna et al. 2014; Popov et al. 2016; Ubhi et al. 2012). Details of the problems and the computational approaches employed are, however, rarely discussed explicitly. Typically, standardized procedures of the early preprocessing steps of the MS data analysis (peak picking, peak alignment and feature linking) are described in some depth, but the subsequent computational approaches employed to suppress unwanted variation is only vaguely outlined. In many cases the employed remedies are procedures concealed within the supplementary material, often involving either principal component analysis (PCA), partial least square discriminant analysis (PLS-DA) or batch removal methods already included in various R packages (Brunius et al. 2016; Leek et al. 2016; Ritchie et al. 2015). Thus, there is an apparent need for automated and robust computational pipelines that can be supported by rational arguments and that can be validated to offer reliable suppression of the unwanted experimental and technical variability.

In summary, as a first step towards making LC–MS based in vitro metabolomics well established and broadly applied, work towards best-practice computational procedures is required, which can help circumventing or compensating for the experimental pitfalls and challenges associated with this emerging methodology including contaminants, carry over effects, intensity decay, and unwanted variability and biases like batch effects. In the work reported here, we performed a pilot study to introduce such a computational pipeline while illustrating the main pitfalls and challenges. The pilot study was designed as a metabolomics setup wherein the non-cancerous mammalian gland derived cell line MCF-10A was exposed to 27 substances that can be divided into four pharmacological classes and a set of pesticides. For each substance a cell lysate extracted 24 h after exposure was analyzed using a modern high resolution LC–MS system. The experimental goal with this setup was to assess whether pharmacological classes would appear as class characteristic metabolic profiles. Based on pitfalls and challenges encountered in the subsequent data analysis, a computational pipeline was established to handle commonly encountered complications including those mentioned above.

In the following we present this workflow in detail including well-founded motivations for each computational step performed. Experimental results are presented showing that this new pipeline offers important contributions towards more reliable in vitro metabolomics studies, based on a state-of-the-art high resolution LC–MS system. In particular, the data after the computational post-processing of the MS spectra show that the cells exposed to the group of anti-inflammatory drugs have a distinct change in their metabolomic pattern compared to the remaining cells including controls.

## Materials and methods

### Chemical compounds

The 27 chemical test agents used in the study are listed in Table 1 and can be divided into either of four pharmacological classes or a set consisting of six pesticides. All compounds were purchased from a local Sigma–Aldrich supplier (Stockholm, Sweden) and dissolved in DMSO with a DMSO final concentration equivalent to 0.01% or less.

**Table 1:**
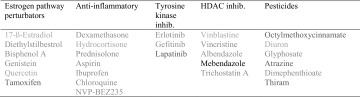
The four pharmacological drug/agent classes with their corresponding compounds used in this study, plus a separate set of six pesticides

The compounds have been color coded as for which batch they were prepared in, orange being one, green two, blue three and red four. In order to retrieve more information regarding batch effects, cells exposed to Mebendazole were placed in all batches giving a total of 12 biological replicates

### Cell line model

The immortalized, non-cancerous MCF-10A, derived from normal human mammary gland, was used as a cell line experimental model. The cell line was obtained from the American Type Culture Collection (ATCC, http://www.atcc.org, Manassas, VA).

### Experimental design

The cells were grown in MEBM supplemented with MEGM Single Quots and cholera toxin (Lonza, Basel, Switzerland) on cover glasses placed inside petri dishes (Thermo Fisher Scientific, Nunc^™^, 8.8 cm^2^). After reaching ∼70% confluence, equivalent to ~2 million cells, the cells were exposed to one of the 27 compounds listed in Table 1 for 24 h. Three biological replicates were created in order to catch biological variation. After 24 h incubation in presence of test agents, cells were harvested. Cover glasses were carefully lifted from the bottom of the petri plates using tweezers and then dipped into the stirring water of the washing unit to rinse both sides of the cover glasses. They were then quickly placed in the quenching solution (−20 °C 80% methanol) followed by detachment of cells using a rubber-tipped cell scraper (Sarstedt, Nümbrecht, Germany). Detached cells were transferred to 1.5 ml conical centrifuge tubes and centrifuged for 10 min at 21,000×*g* 4 °C. The supernatants collected were freeze dried using centrifugal vacuum concentrator (1–2 h).

The whole sample preparation procedure was split into four weeks, creating four sample batches. Each batch contained three Mebendazole replicates (to catch batch effects), three control samples (with three replicates each) being cells treated with only 0.01% DMSO and six blank samples, containing no cells or drugs, only DMSO.

### Mass spectrometry analyses

The freeze dried samples were dissolved in 5% methanol, 0.1% formic acid (FA) and 94.9% deionized MilliQ water, vortexed for 10 s and 20 μl was transferred to a clean tube to produce a pool containing all samples (quality control (QC) samples) for performance monitoring. The samples were analyzed in a constrained randomized order where samples were divided into three blocks, containing one of the three replicates per sample (the 12 Mebendazole replicates were distributed equally between these blocks). The blocks were analyzed sequentially, with a randomized injection order within the blocks where each sample was injected twice. Blank samples, were distributed throughout the analysis to catch contaminants and carry over effects. The analysis was performed on a Thermo Ultimate 3000 HPLC and Thermo Q-Exactive Orbitrap mass spectrometer. The 20 µl of sample was injected to a Thermo Accucore aQ RP C18 column (100 × 2.1 mm, 2.7 µm particle size). The analytical gradient was initialized with an isocratic flow for 3 min (0% B) followed by 5 min (0–20% B), 6 min (20–100% B), 3 min (100% B), 2 min (100% C) and lastly re-equilibration of column for 6 min (0% B), where A is 0.1% FA, B is 89.9% acetonitrile, 10% isopropanol and 0.1% FA and C is 100% methanol, at a flow rate of 0.4 ml/min. Mass spectrometry data were acquired in profile and positive ion mode, using a mass range of 130–900 m/z with a 70 000 FWHM resolution, AGC target 1e6, maximum injection time 200 ms, spray voltage of 4 kV, capillary temperature 350 °C, arbitrary units of sheat gas 30 and auxiliary gas 10.

### LC-MS processing

The acquired raw data was converted to an open source format (.mzML) by *msconvert* from ProteoWizard (Chambers et al. 2012) and preprocessed using the following pipeline within the OpenMS platform (Sturm et al. 2008): The raw data was centroided (peak picking) using *PeakPickerHiRes* (Weisser et al. 2013) and the features (possible metabolites) were quantified by *FeatureFinderMetabo* (Kenar et al. 2014). The parameters with non-default values can be found in Supplementary Table 1. The resulting features were linked across the samples using *FeatureLinkerUnlabelledQT* (Weisser et al. 2013), allowing 15 s retention time tolerance and 5 ppm mass deviation (the linking was performed irrespective of charge state across the samples). The preprocessed data was then further loaded into the statistical software environment R v 3.2.1 (R Core Team 2015), where features without established charge were removed. The processing pipeline for suppressing contaminants, carry over effects and intensity decaying was further implemented in R and the implementation of OOS-DA (optimal orthonormal system for discriminant analysis) was done in MATLAB (R2015a, The MathWorks, Inc., Natick, MA) and used to process the 3803 features remaining after the preprocessing procedure. *prcomp* in R package *stats* was used with default settings to perform principal component analysis (PCA) for visualization of the data in 2D and 3D plots (missing values were replaced by zeros before PCA). The data was always log2 transformed before PCA.

## Results

### A computational pipeline for suppression of experimental variability

**Fig. 1:**
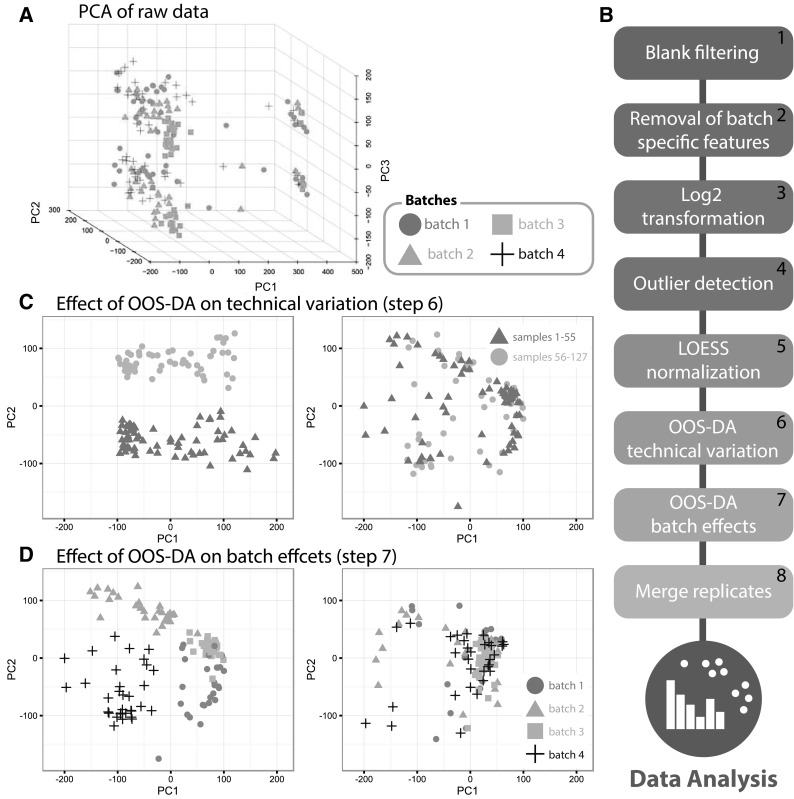
A data processing pipeline to remove technical variability and biases. **a** Initial visualization of the log2 transformed raw MS spectra using PCA for compression of each 3803-dimensional MS spectrum (sample) into 3D. The samples are split into two well separated clusters along the PC3 dimension, a substructure that is not expected to exist. The samples color coded according to batch identities also reveals existing batch effects within the data. **b** An overview of the introduced pipeline including the novel use of the method OOS-DA. **c** Effect of removing the technically related variability associated with the MS analysis using OOS-DA, visible in subfigure **a**. The *left panel* shows the data before removal (where the previous steps in the pipeline (step *1–5*) have been applied), whereas the *right panel* depicts data after removal (step *6*). **d** Effect of removing the batch effects by application of OOS-DA on batch identities, where the *left panel* shows the data before removal (step *1–6*) and the *right panel* shows the data after removal (step *7*)

For this particular pilot experiment and the resulting MS data, a few issues in the data were immediately identified, as revealed by PCA. In Fig. 1a, the result of PCA after compression from 3803 spectral features along the directions of the largest variability (corresponding to PC1, PC2 and PC3) is presented. This plot immediately reveals remarkable and unexpected substructures present in the data that may be described as two major clusters separated along the PC3 dimension as well as undesirable batch effects visual by mean of color coding in Fig. 1a. A comparison with the injection order clarifies that this separation is associated with a particular time point in the MS analysis (A 3D plot visualizing the samples labeled with their corresponding number in the injection order can be seen in Supplementary Fig. 1). This shift could for example be associated with refilling of the mobile phases or changed external conditions which unfortunately were not noted during the experimental execution.

Prior to any further analysis or downstream interpretation of the data, the undesired biases shown in Fig. 1a must be removed. In Fig. 1b, the pipeline for handling this as well as other key issues such as contaminations, carry over effects, and intensity decaying is summarized.

#### Blank filtering and removal of batch specific features

In the first step in Fig. 1b, referred to as *Blank filtering*, blank samples (containing only DMSO, no cells) are being used to detect contaminants. Features that have a median intensity across the blank MS spectra higher than 1% of the maximum intensity of the other samples are labeled contaminants and removed. In the second step of the pipeline illustrated in Fig. 1b, features considered to be batch specific were removed.

#### Log2-transformation, outlier detection and normalization through LOESS

In order to reduce the dynamic range, each MS feature value x (intensity) was replaced by its log 2 value log_2_(x). Potential sample outliers were subsequently detected by calculating the total ion count (TIC) of each sample and visualizing the TIC distribution in a boxplot. Samples with an abnormally low TIC (less than 70% of the average TIC) were removed from the study.

In order to suppress deviation across replicates, an individual MS spectrum was only kept if at least one of the corresponding replicates was sufficiently similar to this particular spectrum. The similarities between pairs of MS spectra were quantified in terms of Pearson’s correlation. Thus a spectrum was kept if the largest Pearson’s correlation coefficient obtained when compared with each of the two other replicates separately was larger than 0.8.

Finally, to account for intensity decaying and suppression, normalization was performed using the R-function *normalizeCyclicLoess* from the limma R-package (Ritchie et al. 2015). This approach allows for normalizing each column (spectrum), iteratively applying LOESS (locally weighted scatterplot smoothing) normalization to normalize each pair of columns to each other.

#### Accounting for batch effects and various technical effects using OOS-DA

In order to achieve more tailor made removal of undesired variation and batch effects in a multivariate dataset, supervised learning methods are required. PLS-DA is a supervised method commonly applied to spectral analysis, while PCA is an unsupervised alternative, likewise previously used in this context (Brereton and Lloyd 2014; Ganna et al. 2014). PCA and PLS-DA both support designing latent variable models that can help removing batch effects associated with a subset of the latent variables extracted (See Supplementary Fig. 2 for demonstration of result). An obvious disadvantage of unsupervised approaches, such as PCA, is that no batch (class) information can be used to select the latent variable models. Thus, supervised approaches like PLS-DA are inherently more efficient. Since PLS-DA originates from linear regression rather than linear discriminant analysis (Brereton and Lloyd 2014), we preferred a theoretically more natural (well founded) method developed by Okada and Tomita (1985), in the following called OOS-DA (optimal orthonormal system for discriminant analysis). This method may be viewed as a generalization of Fisher´s linear discriminant. OOS-DA has been used previously to design a new type of classification method where a nearest neighbor classifier is applied in the 3-dimensional latent variable space produced by OOS-DA (Darmanis et al. 2011). Employing OOS-DA in our case results in a latent variable model which for an MS spectrum **x** can be expressed as **x** ≈ **Pt** where the vector **t** contains the values of the latent variables and the columns of the matrix **P** are known as loading vectors. By designing the model (loading) matrix **P** based on the batch identities of each sample, a compressed latent variable model that captures the main variability in the MS spectra associated with batch is obtained. After determining a suitable dimensionality of this model, the batch effects are suppressed by removing these dimensions from each sample, yielding new spectral vectors **x**_new_ as **x**_new_ = **x** − **Pt**.

In this work, OOS-DA modeling was implemented in MATLAB and used to remove the evident subdivision of the data into two parts occurring between sample number 55 and 56 in the injection order shown in Supplementary Fig. 1.

In order to avoid trivial problems with close to singular scatter matrices in the OOS-DA algorithm in the computational pipeline, a pre-processing step based on PCA is used to compress the original spectra into feature vectors of lower dimensionality, which are instead used as inputs to OOS-DA. The feature vectors are selected to have N−k dimensions where N is the number of samples and k is chosen to make the scatter matrices numerically non-singular. Thus the latent variable models created by means of OOS-DA correspond to score vectors of dimension N−k and the batch removal (subtraction) is performed in this reduced space. After the removal of the undesired variation, the pipeline transforms each cleaned score vector back to the original spectral space by means of the loading vectors already obtained from the PCA. In order to employ OOS-DA for the removal of the experimental shift (step 6 in the pipeline), the spectra were compressed to N−2 dimensions (k = 2) and two classes were defined. The samples 1–55 belonged to the first class and the remaining samples, 56–127, to the second class. The OOS-DA model was then employed to obtain a sequence of latent variable models designed to discriminate between the two classes (batches) as well as possible, according to the built-in criterion of the OOS-DA algorithm. The most suitable number of latent variables to use in order to suppress the technical variability was determined by automatic tuning, described in the following section. The same procedure was then employed once more (step 7 in the pipeline) to remove the batch effects by assigning each sample to one of four classes (their batch identities), this time performing the PCA based pre-compression using k = 8. The effects of these actions are illustrated in Fig. 1c, d.

To avoid the introduction of spurious features, the positions of missing values in the spectra were saved before application of OOS-DA, and then missing values were reintroduced again at these positions before merging the replicates by replacing them by their median value.

### Tuning the dimensionality of the latent space in OOS-DA modeling

An inherent hurdle of OOS-DA modeling (and similar approaches like PLS-DA) for removal of undesirable experimental effects lies in the selection of the number of latent dimensions. To avoid error prone manual tuning, parameters were selected automatically, based on replicates of the same sample being continuously injected in the injection order, as well as equally distributed in the sample preparation batches. More specifically, the number of latent variables to be removed was determined by the following idea: Select the best latent space dimensionality d_best_ by looking at the Mebendazole replicates which were present in all batches. After subtraction of the latent dimensions, the Mebendazole replicates should cluster more tightly. In other words, the geometrical spread of the Mebendazole replicates should decrease. This was quantified and optimized by first defining the Mebendazole centroid for class (batch) *k* as

1 $${\overline m _k}=\frac{1}{{{N_k}}}~\sum\limits_{{{\overline x }_n}\varepsilon class~k} {{{\overline x }_n}}$$

where $\overline {{x_n}}$ denotes one of the Mebendazole spectra belonging to class *k* and *N*_*k*_ denotes the total number of Mebendazole spectra in this class. Then as a global measure of spread we used the average Euclidean distance b between the within-class Mebendazole centroids and the global centroid defined as

2 $$b=\frac{1}{K}\mathop \sum \limits_{k=1}^K {\mathop {\overline m }\limits{} _k} - \mathop {\overline m }\limits^{} ~$$

where K denotes the total number of classes and the global mean is calculated as

3 $$\mathop {\overline m }\limits^{} =\frac{1}{K}\mathop \sum \limits_{k=1}^K {\mathop {\overline m }\limits{} _k}$$

The distance measure used to calculate ||**z**|| for any **z** is the Euclidean distance. Using these definitions, the optimal dimension d_best_ was selected as the smallest dimension where the distance b(d) stabilizes after an initial drop when viewed as a function of the dimension d, see Supplementary Fig. 3. More specifically, d_best_ was selected as the smallest dimension where the relative change in b(d) is less than 1%. Thus d_best_ was the smallest value that was satisfying the inequality |b(d_best_ + 1) − b(d_best_)|/b(d_best_) < 0.01.

For each conceivable dimensionally d, b(d) was calculated for the defined Mebendazole classes. Supplementary Fig. 3 shows a plot of b(d) for the case of the injection order effect mentioned earlier.

### Experimental validations

**Fig. 2:**
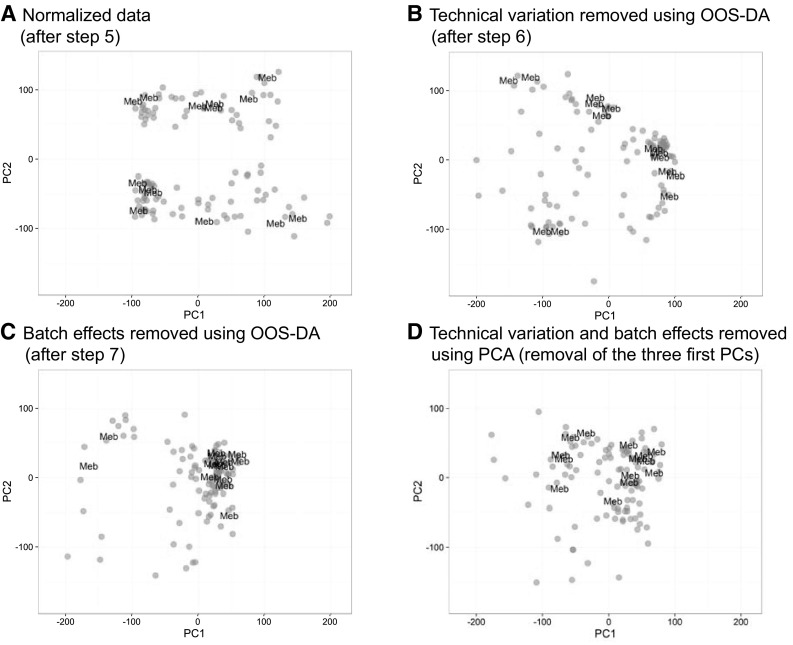
Reduced geometrical spread for the drug Mebendazole after application of OOS-DA modeling. The same score plots are presented as in Fig. 1c, d but coded to visualize the many Mebendazole exposed replicates (labeled as “Meb”). These samples are distributed across the injection order and in all four batches. **a** The Mebendazole samples, without removing the unwanted variation from the data (only step *1–5* from the pipeline illustrated in Fig. 1b were applied). **b** The Mebendazole samples after removing the technically related variability using OOS-DA (step *6* in the pipeline). **c** The Mebendazole samples after removing the batch effects using OOS-DA (step *7* in the pipeline). As expected and desired, after the removal of unwanted variation, the Mebendazole exposed replicates are clustering more tightly to each other (same scale on the axes in all subfigures). **d** The Mebendazole samples after removing the first three principal components and thereby removal of the most dominant variation. The Mebendazole samples are closer together, but still separated by the shift

To confirm that the technical variability and the batch effects were suppressed by means of the OOS-DA based modeling described above, the positions of the Mebendazole replicates, before and after processing, were visualized in PCA score plots, Fig. 2.

#### OOS-DA for finding biological variation of interest and permutation tests for determination of level of significance

As demonstrated above, OOS-DA is able to target specific variation based on labeled training examples reflecting this variability. In the same way that this feature of OOS-DA can be used to target unwanted variation for removal of technical variability (like batch effects), it can also be used to directly target the variation/separation of real interest, meaning the separation between the actual classes one is interested to discriminate between (drug subclasses in our case). This idea to use OOS-DA to directly focus on the variability of real interest, rather than first removing technical variability, was demonstrated in a previous article (Darmanis et al. 2011). There OOS-DA was used to directly compress high dimensional molecular (protein) data into a 3-dimensional space by sifting out those dimensions in the original data space where the pre-defined subclasses in the dataset separate as well as possible. If the main goal is to achieve acceptable discrimination between the subclasses, such a direct approach might be sufficient. However, OOS-DA may be used twice, first to remove technical variability and then in order to focus on the subspace offering the best subclass discrimination. This might give improved separation of the biological subclasses of interest and improved spectra being less contaminated by the technical variability.

**Fig. 3:**
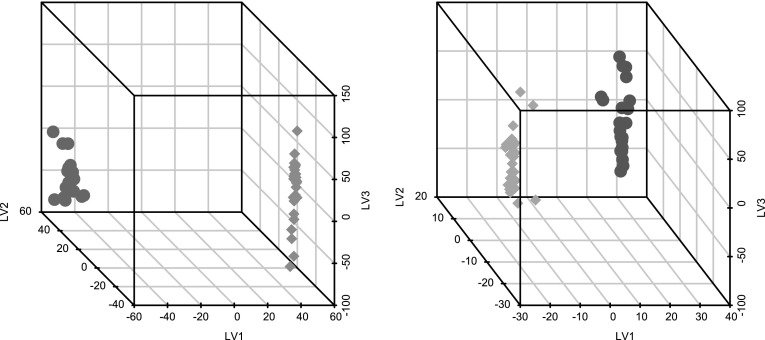
Separations achieved by OOS-DA modeling with correct and randomized class assignments. Each point in these 3D plots is represented by its coordinates in the latent variable space created by the OOS-DA modeling (LV1 denotes latent variable 1 etc). As shown, methods like OOS-DA will always be able to find a separation between any classes defined in high dimensional data. The large and clearly visible separation achieved with the correct classes (**left**) is not very outstanding compared to the also large and clear separation achieved with the randomized classes (**right**)

One fundamental pitfall associated with dimensionality reduction of data having many dimensions/features is the risk of finding random patterns rather than something that corresponds to true differences and similarities. In particular, as there are many features, there will regularly be a perfect (non-overlapping) separation possible between any class definitions employed (Kjeldahl and Bro 2010; Kohler et al. 2016). This can be illustrated using our own data by randomizing the class assignments and performing the OOS-DA modeling on these random classes, as illustrated in Fig. 3. In the left panel, data are presented for the three main latent variables extracted using OOS-DA employing the true labels (control samples versus drug exposed samples) whereas the right panel shows the corresponding result for a random assignment of class labels. Apparently OOS-DA is able to find low-dimensional subspaces that show clear separations between the assigned classes in both cases.

In order to quantify how easy it is to get this kind of separation shown in the left part of Fig. 3 for our dataset by random chance, we defined and employed the separation score s_sep_ = b/w, as defined in (5) using the definition of b introduced in (1–3) and the definition of w in (4). Thus *b* denotes again the average distance between class centroids and the global centroid, *w*_*k*_ is the average distance between the centroid ${\overline m _k}$ of class *k* and the samples belonging to this class, and *w* is the average within-class distance from a sample and its class centroid. In brief *s*_*sep*_ is a ratio quantifying between-class versus within-class variability.

4 $$w=\frac{1}{K}\mathop \sum \limits_{k=1}^K {w_k}~~~~~~~where~~~~~~~{w_k}=\frac{1}{{{N_k}}}\mathop \sum \limits_{{{\overline x }_n}\varepsilon class~k} {\overline x _n} - {\overline m _k}$$

5 $${s_{sep}}=\frac{b}{{\frac{1}{K}\mathop \sum \nolimits_{k=1}^K {w_k}}}=\frac{b}{w}~$$

**Fig. 4:**
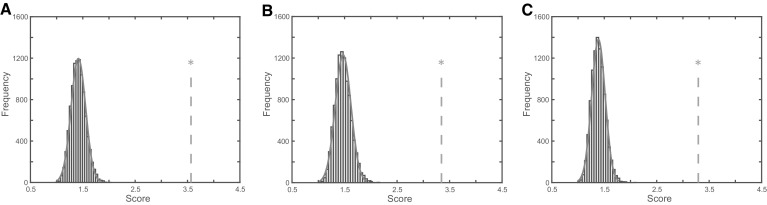
Permutation results for controls versus the rest. The three histograms showing the distributions of 10,000 separability score values achieved by extracting the drug subclass variation of interest using OOS-DA. This was done when **a** the data still contained the unwanted batch/shift related variation (after step *5* in the pipeline in Fig. 1b), **b** the unwanted variation had been suppressed by means of OOS-DA based batch/shift removal and **c** the unwanted batch/shift induced variation had been suppressed by removal of the first three principal components of the dataset. From **a** one may conclude that direct application of OOS-DA without any attempt to perform batch removal is quite successful at ignoring the unwanted variation, yielding a significantly higher separability score than achieved when using random permutations. The results in **b** show again a statistically significant score value although it is lower than in **a**. Finally the results in **c** show that batch removal using unsupervised PCA, instead of using OOS-DA, yields a lower separation score which is still statistically significant

In order to demonstrate the ability of OOS-DA to directly find class differences of interest in our metabolomics dataset (without any attempt to remove technical variability as a pre-processing step), samples of control versus treated cells were studied. As depicted in Fig. 4a, this direct use of OOS-DA was able to suppress (ignore) the unwanted shift and batch effects, while keeping a substantial part of the desired biological variation (drug class information), reflected by the separability score value s_sep,correct_ = 3.57. To assess the level of significance of this score value, it was compared to the score values achieved when performing random permutations of the biological class labels. The random permutations of class assignments were performed 10,000 times, followed by OOS-DA modeling and subsequent calculation of the separation score s_sep_ for each model in the resulting latent 3-dimensional space obtained by compressing all spectra. Finally the resulting distribution of permutation generated separation score values were compared to the separation score obtained when using the correct class assignments.

To study if removal of unwanted shift and batch variation using OOS-DA would further increase the desired biological separation as suggested above, the same procedure was applied to the data processed by the pipeline illustrated in Fig. 1b. The results are presented in Fig. 4b showing that OOS-DA is able to significantly separate (p-value < 0.0001) the two classes also in this case, but the score value s_sep,correct_ was decreased from 3.57 to 3.35. Thus the attempt to remove shift and batch variation using OOS-DA did not improve the separability in this case. However, still this score value is larger than the score 3.29 obtained when PCA is used instead of OOS-DA for the batch removal step, as illustrated in Fig. 4c. Notably the PCA based removal (which is unsupervised and does not rely on any batch information, thus only taking away the dimensions having the largest variability) yields almost as good separation score as when using the supervised OOS-DA. This suggests that the current dataset contains a lot of batch independent technical variability. This is one possible explanation why batch removal using OOS-DA did not yield the highest separation score here. Perhaps the attempt to remove shift and batch effects using OOS-DA made some part of the batch independent experimental noise enhanced sufficiently much to yield the observed decrease of the separation score from 3.57 to 3.35.

**Fig. 5:**
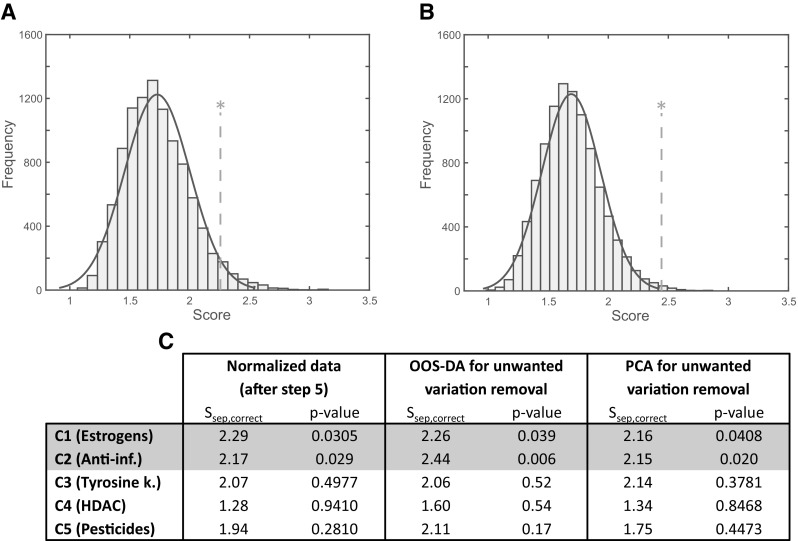
Permutation results for one drug class (estrogen pathway perturbator, anti-inflammatory, tyrosin kinase inhibitors, HDAC inhibitors, and pesticides) versus the rest. OOS-DA was used as in Fig. 4, to discriminate a single class from all other samples. **a** After removal of unwanted variation using OOS-DA the estrogen pathway perturbators resulted in s_sep,correct_ = 2.26 (p-value = 0.039) and **b** the anti-inflammatory drugs in s_sep,correct_ = 2.44 (p-value = 0.006). **c** Table summarizing a comparison of separation scores for all classes before and after trying to remove unwanted variation using OOS-DA and PCA, respectively

In addition to studying separation between control and treated cells, the same procedure as used to produce Fig. 4 was also used to study how well each of the five pharmacological groups separate from the remaining samples. Figure 5a, b present the two most outstanding classes together with a table in Fig. 5c presenting scores obtained with different data processing approaches for all the five pharmacological groups. According to the table, the anti-inflammatory drugs and the estrogen pathway perturbators yield separation scores which are statistically significant already after step *5* in the pipeline. After removal of unwanted variation by means of OOS-DA, the anti-inflammatory drugs separation score is increased from 2.17 (s_sep_ after step *5*) to 2.44 with a p-value of 0.006, while the estrogen pathway perturbators maintain a similar separation score at a similar significance level. Using PCA for removal of the unwanted variation lowers the separation score for the estrogen pathway perturbators, while the score for the anti-inflammatory class is mostly unaffected.

## Discussion

Metabolomics has over the recent past shown its potential in various fields including in vitro systems pharmacology (Aftab et al. 2014). Utilizing an information rich measurement technique like MS, following a separation process like liquid chromatography, one can acquire low molecular weight agents from highly complex mixtures. LC–MS based metabolomics is a powerful and competitive approach which unfortunately is coupled to multiple pitfalls, which can make it challenging to use. A carefully planned experimental design procedure is therefore essential in order to retrieve reliable biological information (Kohler et al. 2016). However, in many cases it will be necessary to divide samples into smaller batches. Especially in larger studies, which are becoming much more common as large computational resources get more accessible through cloud providers.

Therefore, as a key step towards qualifying LC–MS based metabolomics as a robust and reliable methodology, we have devised a computational pipeline to manage pitfalls and challenges typically attached to this emerging technology. The experimental results obtained suggest that our computational pipeline is able to successfully and drastically suppress spurious data read-out inherent in the current methodology and instrumentation. Especially with a more robust and faster experimental setting, relative to that described here, the pipeline holds strong promise to enable a sensitive and yet robust method for large-scale metabolomics. However, worth having in mind is the inescapable trade-off between removing experimental variability in data and retaining the biological information of interest. In Fig. 2c, there are two Mebendazole replicates that are not merged with their fellow replicates. If merged by removing a larger dimensionality of the latent space than proposed by the parameter tuning, the replicates were united with a consequential loss in s_sep,correct_ (data not shown).

Multiple tools for data management of metabolomics data have already been developed and are freely available through sharing on source code browsers like Github and Bitbucket or through data analysis platforms like KNIME Analytics (Berthold et al. 2007), OpenMS (Sturm et al. 2008) or workflow4metabolomics (Giacomoni et al. 2015). However, finding the most appropriate tool for the task can be rather challenging and many tools often lack detailed documentation. In this work we introduce a new method suitable for the metabolomics field, here called OOS-DA, which can be used to target and remove specific multi-dimensional structural variability of interest like batch effects, as well as directly compressing data into a small set of class information rich features (Darmanis et al. 2011). It comes with a tailor made permutation test, presented here for the first time, which makes it possible to determine if the actual separations obtained in the low-dimensional space produced by OOS-DA is statistically significant (or easily obtained also for randomly assigned class labels). To help potential future users, we present OOS-DA and its associated permutation test together in the context of a typical user case.

More specifically, we believe that this work provides the following main contributions:

- Provides a proof-of-principle example demonstrating that an LC–MS based metabolomics approach is able to yield metabolite profiles that contain discriminatory information with respect to chemically induced and drug class specific effects at the metabolite level.
- Identifies, describes and provides successful computational methods to compensate for several key pitfalls and challenges in LC–MS based metabolomics.
- Introduces and successfully employs a theoretically motivated computational pipeline for suppression of experimental variability and biases, within the aforementioned area.
- Introduces for the first time in the context of metabolomics a latent variable modeling method, denoted OOS-DA, that can be used in two different ways: (1) To subtract away undesired technical variability that can be specified by the user. (2) Make a latent variable model that will keep as much as possible of the real class information of interest in the resulting low dimensional latent variable space.
- Provides additions to the original OOS-DA method suitable for the current and similar applications in the form of: (1) An algorithm for automated selection of the dimensionality of the latent variable space removed. (2) A permutation test used to evaluate if the class separations obtained in the latent variable space are statistically significant or not.

Regarding the experimental findings, the anti-inflammatory and the estrogen pathway perturbators agents were found to generate the most prominent changes in the collected metabolomic profiles (s_sep_ = 2.44 and s_sep_ = 2.26) while the set of pesticides did not show any significant changes. Moreover, the HDAC inhibitors and the receptor tyrosine kinase inhibitors resulted in even lower and less significant separation scores. This low separation score for the receptor tyrosine kinase inhibitors may perhaps be explained by the low number of members of this class. Out of 49 samples (27 agents and 22 controls) only three belong to the class of receptor tyrosine kinase inhibitors. Therefore the probability of randomly picking three members from any of the larger classes will be very high. Regarding the HDAC inhibitors, this class of drugs has antiproliferative effects and induces cell cycle arrest in general. The fact that our results only show small differences to other classes and controls, is part in line with previous findings demonstrating that the HDAC inhibitor Trichostatin A stabilizes part of the metabolome in vitro and that treatment effects are more pronounced with longer exposure times (96 h), compared to the 24 h exposure time used here (Ellis et al. 2010).

In conclusion, LC–MS based in vitro studies of chemically induced changes of the metabolome has a strong potential provided one is employing robust experimental protocol settings in combination with the kind of computational pipeline introduced here, which can specifically suppress experimental variability and biases.

## Electronic supplementary material

Below is the link to the electronic supplementary material.

Supplementary material 1 (PDF 114 KB)

## Abbreviations

LC: Liquid chromatography
MS: Mass spectrometry
PCA: Principal component analysis
LVX: Latent variable number X
PCY: Principal component number Y
OOS-DA: Optimal orthonormal system for discriminant analysis
PLS-DA: Partial least squares for discriminant analysis
NMRS: Nuclear magnetic resonance spectroscopy
