# Supplementary material for: Mass spectrometry based metabolomics for in vitro systems pharmacology: pitfalls, challenges, and computational solutions
Source: Metabolomics. 2017 May 19;13(7):79. doi: 10.1007/s11306-017-1213-z (PMC5438430; doi:10.1007/s11306-017-1213-z)
Supplement: Supplementary file 1 — Supplementary material 1 (PDF 114 KB) [file 11306_2017_1213_MOESM1_ESM.pdf]

# Supplementary Material

## Mass spectrometry based metabolomics for in vitro systems pharmacology: Pitfalls, challenges, and computational solutions

Stephanie Herman, Payam Emami, Obaid Aftab, Shibu Krishnan, Emil Strömbom, Rolf Larsson, Ulf Hammerling, Ola Spjuth, Kim Kultima*, Mats Gustafsson*

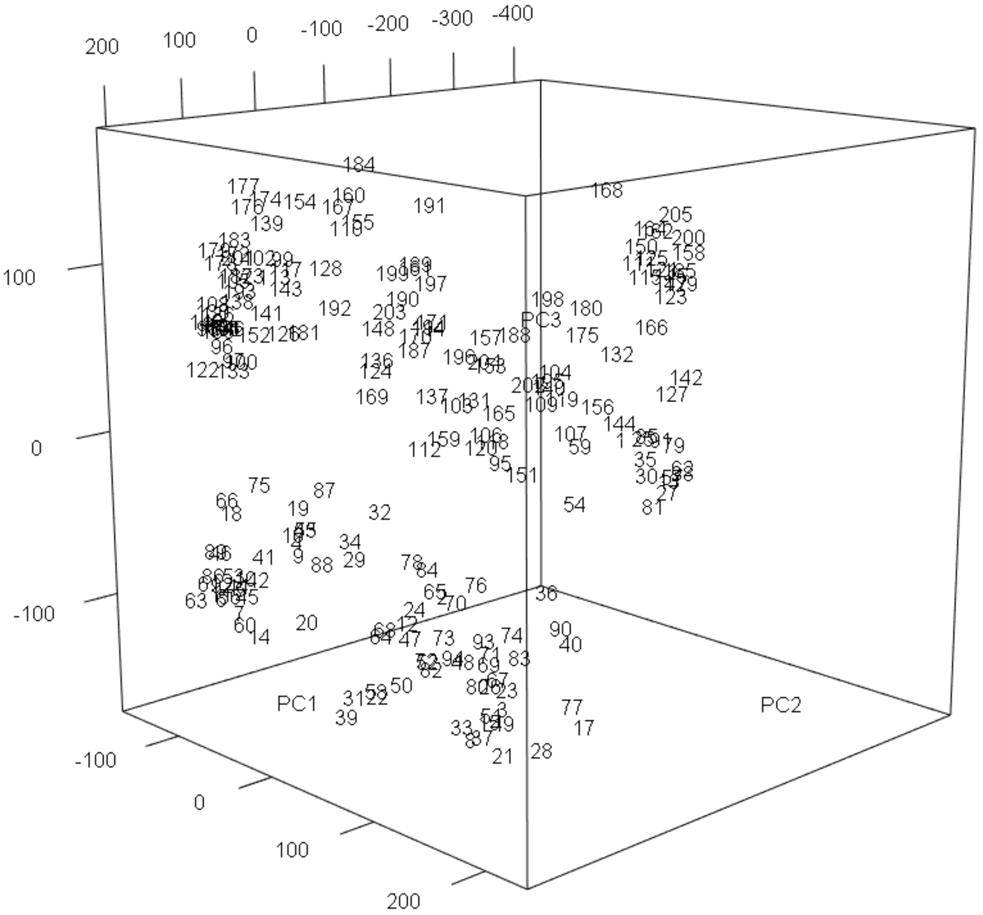


**Suppl. Fig. 1 Initial visualization of the MS spectra labeled according to the injection order.** PCA was used for compression of each 3803-dimensional MS spectrum (sample) into 3D which was labeled with their corresponding number in the injection order. The plot shows that the two clusters seen in Fig. 1A are related to the injection order.

**Suppl. Table 1 All parameters with non-default values used in OpenMS.** For all parameters not mentioned, the default values were used.

| Parameter | Value |
|---|---|
| *FeatureFinderMetabo* | |
| noise_threshold_int | 5000 |
| Chrom_peak_snr | 3 |
| Chrom_fwhm | 3 |
| Mass_error_ppm | 5 |
| Trace_termination_outliers | 3 |
| Min_sample_rate | 0.7 |
| Min_trace_length | 1 |
| Max_trace_length | 35 |
| Width_filtering | auto |
| Local_rt_range | 30 |
| Local_mz_range | 6.5 |
| Charge_lower_bound | 1 |
| Charge_upper_bound | 10 |
| Use_smoothed_intensities | false |

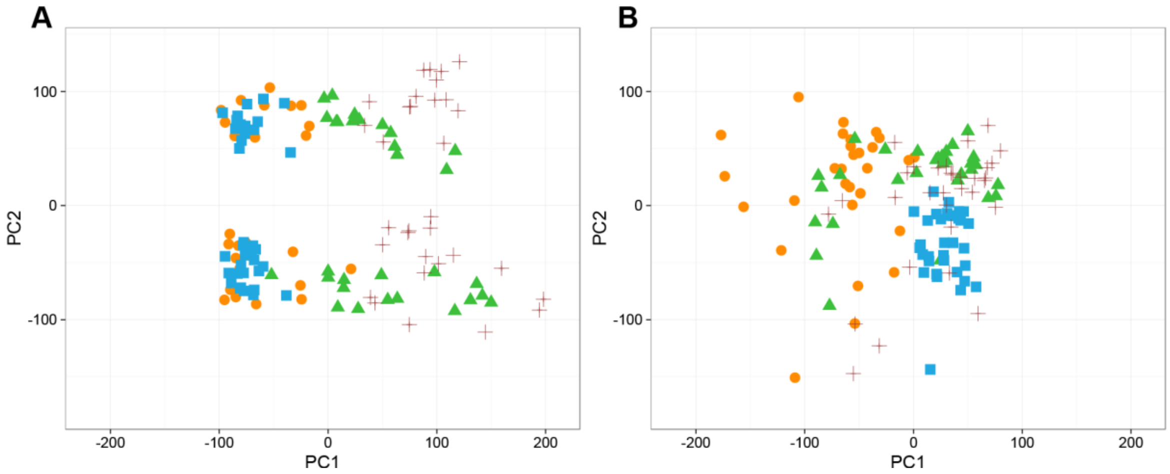


**Suppl. Fig. 2 Removing the unwanted variation using PCA.** PCA was performed on the data visualized in Fig. 4A. The data was further rebuilt, excluding the three first principal components (PC1, PC2, PC3) and thereby removing the most dominant variation in the data. The PCA plots demonstrate the effects of removing the unwanted variation using PCA, where the left panel **(A)** show the data before removal and the right panel **(B)** show the data after removal. The samples have been shape- and color-coded according to batch identities. Conclusively, subfigure B indicates that the unwanted variation seems to have been fairly successfully removed, the batches have moved closer together. However, looking at Fig. 2D, we see that the shift caused by technical variation is still present; the Mebendazole replicates are still split into two groups.

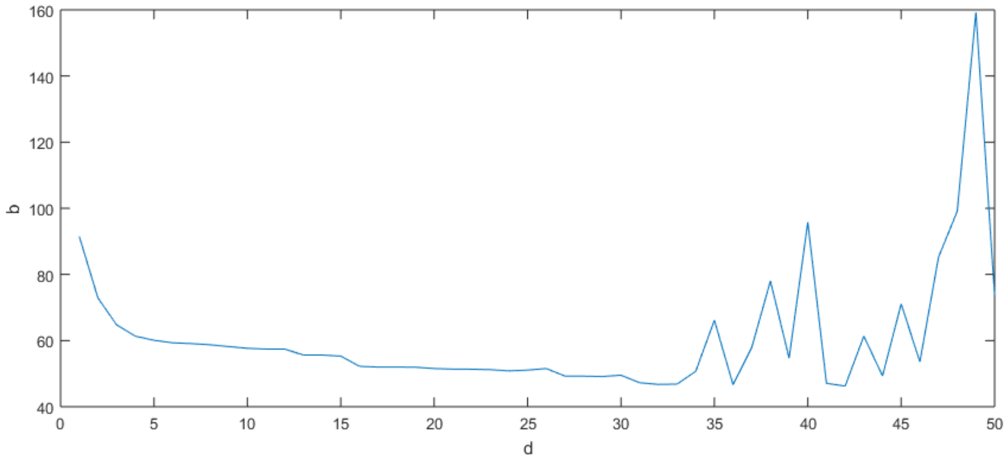


**Suppl. Fig. 3 Automated selection of the latent space dimensionality during OOS-DA based suppression of undesirable experimental effects.** For each conceivable dimensionality d, the spread b(d) of the Mebendazole spectra is quantified and displayed as a function of d. In the particular case presented here, the suggested number of dimensions is $d_{best}$=6 as the relative change between d=6 and d=7 is less than 1%.
